# Graphene Formation through Pulsed Wire Discharge of Graphite Strips in Water: Exfoliation Mechanism

**DOI:** 10.3390/nano11051223

**Published:** 2021-05-06

**Authors:** Shigeru Tanaka, Daisuke Inao, Kouki Hasegawa, Kazuyuki Hokamoto, Pengwan Chen, Xin Gao

**Affiliations:** 1 Institute of Industrial Nanomaterials (IINa), Kumamoto University, 2-39-1 Kurokami, Chuo-ku, Kumamoto 860-8555, Japan; tanaka@mech.kumamoto-u.ac.jp; 2 Technical Division, Faculty of Engineering, Kumamoto University, 2-39-1 Kurokami, Chuo-ku, Kumamoto 860-8555, Japan; inao@tech.kumamoto-u.ac.jp; 3 Graduate School of Science and Technology, Kumamoto University, 2-39-1 Kurokami, Chuo-ku, Kumamoto 860-8555, Japan; hasegawa@mech.kumamoto-u.ac.jp; 4 State Key Laboratory of Explosion Science and Technology, Beijing Institute of Technology, Beijing 100081, China; pwchen@bit.edu.cn

**Keywords:** pulsed wire discharge (PWD), graphene, high-speed imaging, underwater shock wave

## Abstract

This study aims to clarify the mechanism of exfoliation of graphene through electrical pulsed wire discharge (PWD) of a graphite strip, made by the compression of inexpensive expanded graphite in water. The explosion of the graphite strip was visualized using a high-speed video camera. During the energized heating of the sample, explosions, accompanied by shock waves due to expansion of gas inside the sample, occurred at various locations of the sample, and the sample started to expand rapidly. The exfoliated graphene was observed as a region with low light transmittance. The PWD phenomenon of graphite strips, a type of porous material, is reasonably explained by the change in electrical resistivity of the sample during discharge and the light emission due to energy transition of the excited gas.

## 1. Introduction

Graphene, a two-dimensional (2D) material first prepared by Noveselov et al. [1] in 2004 using the mechanical exfoliation method, has proven to be a futuristic material with numerous applications [2,3,4,5,6,7,8]. Its one-atom-thick graphitic layer leads to various outstanding properties [9], including excellent mechanical properties [5,6,10], ultrahigh optical properties [11,12], high electronic properties [1,4,13], and high thermal conductivity [14,15].

Pulsed wire discharge (PWD), or electrical explosion of wire (EEW), is a vapor phase method used for the synthesis of nanoparticles. In this method, a high-density current from a capacitor discharge is passed through a metal wire (resistive element) which transforms and expands into boiling droplets, heated vapor, or plasma by Joule heating [16]. The instantaneous state change produces shock waves. This phenomenon has been applied to high-density plasma generation technology [17], wire explosion spraying technology using droplet particles of wire [18], initiation technology for energetic materials [19,20], and shock wave generation technology for food processing [21]. The main focus of research on this technology is the production of functional nanopowders. Many studies on PWD have been conducted by varying the elements of the wire, the explosion environment of the wire, and the combination of wires. This technology exhibits high universality in various synthetic studies, including high-quality metal nanoparticles [16,22,23], oxides [24,25], carbides [26], nitrides [27], and alloy nanoparticles [28]. In these studies, a solid wire was used as the electrical resistive element as a rule. PWD and EEW are methods for obtaining nanoparticles by triggering a change of state in a resistive element, but it is quite difficult to recover only graphene among various types of carbon allotropes under a more excited state such as the liquid, gas, or plasma phase. Recently, Gao et al. successfully recovered graphene nanosheets by applying a pulsed current to a high-purity graphite stick. They tuned the current density to melt a part of the graphite, resulting in the loss of van den Waals forces [29]. They applied this technique to expanded graphite (i.e., graphite strips) containing a large amount of nitrogen gas. As a result, graphene nanoplatelets, with a particle size in the range of 100 to 200 μm and a thickness of approximately 3 nm, were recovered under conditions of lower current density [30].

To better understand the formation mechanism of nanoparticles during pulsed wire discharge, various techniques have been used to investigate the Joule heating process, product expansion, shockwave propagation, and plasma formation induced by pulsed discharge. Using a high-voltage probe, Rogowski coil, and oscilloscope, the discharge current and voltage data can be recorded to analyze phase transitions, such as melting, vaporization, and plasma formation [28,31,32,33,34]. These results can be further applied to estimate the energy input during the different processes and investigate the formation mechanism from the standpoint of time-resolved energy. High-speed cameras and flash X-ray high-speed cameras are time- and space-resolved techniques used to visualize the product expansion, shockwave propagation, and plasma formation during pulsed discharge [33]; these observation techniques are beneficial for mechanism analysis and simulation research on pulsed wire discharge. The above techniques have been widely utilized to investigate the pulsed discharge of metal wires and promote their development and applications in nanoparticle production. However, because PWD is a high-speed phenomenon that causes high-intensity light emission, cameras with insufficient time resolution capture the explosions as overexposed images [35,36,37,38]. Furthermore, the observation of a pulsed discharge of graphite materials has been rarely reported, which is owed to the short period of related investigation, inhibiting further development of related research.

In this study, the explosion of graphite strips, heated by a pulsed high current, was observed using a single-wavelength laser source and a high-speed video camera equipped with a band-pass filter. This was used to demonstrate the mechanism of graphene exfoliation proposed in our previous work [30]. The particles produced by this process were characterized using powder X-ray diffraction (XRD). The mechanism of graphene exfoliation by the PWD technique without a resistive state change is discussed.

## 2. Materials and Methods

**Figure 1:**
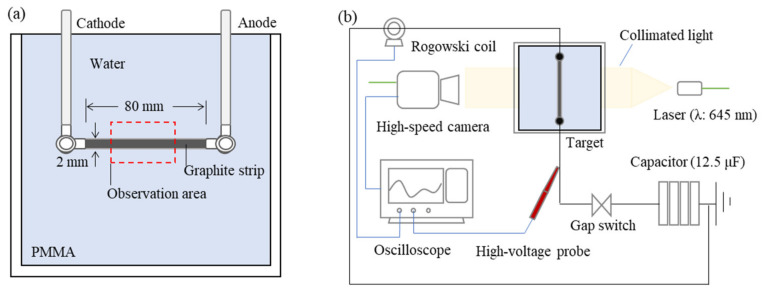
Schematic illustration of the high-speed observation experiment: (**a**) Details of observation target; (**b**) Electrical circuit, instrumentation connections, and the shadowgraph optical observation layout.

Experiments using the shadowgraph technique were conducted to visualize the explosion behavior of graphite strips under pulsed high currents. The details of the visualization target and the shadowgraph layout are shown in Figure 1. The target shown in Figure 1a consisted of a cubic polymethyl methacrylate (PMMA) water tank with a side length of 200 mm, electrodes connected to a capacitor, and graphite strips. The graphite strip was made from expanded graphite (Jinglong Special Carbon Co., Ltd., Beijing, China). First, the graphite flake powder was treated with sulfuric acid or nitric acid, followed by heating in an inert gas medium for expansion. Then, the powder was mechanically compressed to form a graphite paper structure (density: approximately 1.4 g/cm^3^). The cheap inert gas used for the production of graphene was N_2_ gas. The width, thickness, and length of the graphite strips were 2, 0.5, and 80 mm, respectively, and their weight was 0.113 g. The graphite strips were connected to the electrodes in a tank filled with water. The electrical circuit, instrumentation connections, and shadowgraph optical observation layout are shown in Figure 1b. The discharge system used in this study was custom-made by Nichicon Corporation, Kyoto, Japan and its discharge voltage can be adjusted up to 40 kV. The capacitance and the inductance of the capacitor system were 12.5 µF and 3.6 μH, respectively. The discharge voltage was measured using a high-voltage probe (EP-50K, Nissin Pulse Electronics Co., Ltd., Tokyo, Japan) connected to the anode, and the discharge current was measured using a Rogowski coil (current monitor 101, Pearson Electronics, Inc., San Jose, CA, USA) placed on the cathode side. Each waveform profile was recorded using an oscilloscope (DPO7254C, Tektronix, Inc., Beaverton, OR, USA). The Rogowski coils were connected to the oscilloscope via two attenuators (20 dB) connected in series. The discharge voltage *U_R_* (without discharge noise) was obtained (by removing the discharge noise contained in the measured voltage) using the following equation:

(1) $$u_{R}=u_{m}-L\frac{di}{dt}-Ri,$$

where *u_m_* and *i* are the measured voltage and current, respectively, *L* is the inductance of the system (3.6 μH), and *R* is the resistance of the electrodes and partial cable in the measured region (0.1 Ω).

The target, high-speed video camera (HPV-X2, Shimadzu Corporation, Kyoto, Japan), and single-wavelength laser source (Cavilux Smart UHS (50 W), CAVITAR Ltd., Tampere, Finland) were placed on the same axis. The high-speed video camera captured 256 images. The frame rate and exposure time were set to 100 and 50 ns, respectively. PWD is an explosion phenomenon accompanied by shock waves and high-intensity light emission. The camera lens was fitted with a band-pass filter with a center wavelength and half-width of 640 and 12 nm, respectively. The wavelength of the laser was 645 ± 10 nm. The laser point source was converted to collimated light using a plano-convex lens. The exposure time of the camera was synchronized with the laser pulse; this imaging configuration blocks the discharge light emission and images the density changes of water as a shock wave. The trigger signal for the camera imaging was the output from the oscilloscope. The observation area of the explosion phenomenon is shown in Figure 1a. Discharge voltages of 20 and 30 kV were used for the experiments.

To evaluate the particles produced by this method, a collection experiment was conducted. The graphite strips were exploded in a sealed steel container filled with water under the same conditions as the optical observation experiments. The particles recovered from water with a dispersion of fine particles were analyzed by XRD using a Bragg-Brentano diffractometer (Ultima IV, Rigaku Co., Ltd., Tokyo, Japan) with Cu-K_α1_ radiation.

## 3. Results and Discussion

**Figure 2:**
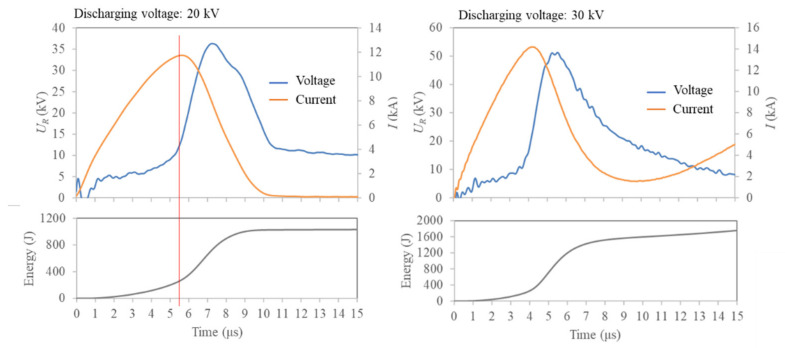
Profiles of discharge voltage, current, and energy injected into the graphite strip.

The profiles of discharge voltage, current, and energy injected to the graphite strip are shown in Figure 2. At a discharge voltage of 20 kV, the voltage and current increases for 5.5 μs after the start of discharge. In other words, the electrical resistance of the graphite strip decreases during this period. As with isotropic graphite materials, the electrical resistivity of graphite strips is dependent on their temperature. The electrical resistivity of the graphite strips decreases up to a temperature of approximately 873 K. The electrical resistivity of the graphite strip at 873 K is 65% of its resistivity at room temperature. After 873 K, the electrical resistivity of the graphite strip starts to increase. The electrical resistivity at approximately 2500 K is 85% of the resistivity at room temperature [39]. The decrease in the electrical resistivity of the graphite strip, that occurred immediately after the start of the discharge, is considered to be in accordance with the temperature dependence of the electrical resistivity. After 5.5 µs, the voltage increases, and the current decreases, which indicates an increase in the electrical resistance. The energy injected into the graphite strip from the start of discharge to 5.5 µs was 260 J. This is much less than the energy required to completely melt the graphite strip (1190 J). Therefore, the graphite strips remained in the solid phase during this time. The same phenomenon was observed at a discharge voltage of 30 kV. However, the electrical resistance started to increase at approximately 4.0 µs.

**Figure 3:**
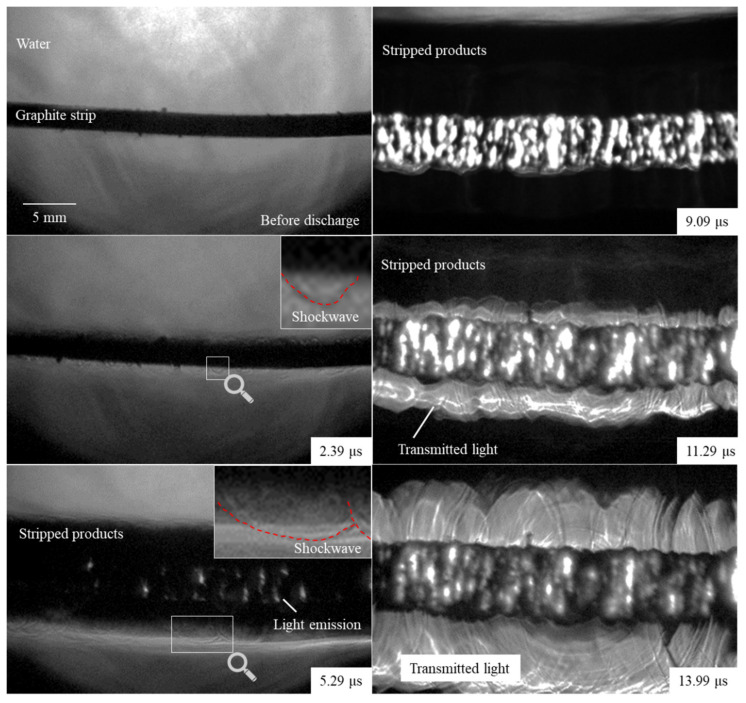
Images of an underwater explosion caused by the rapid heating of a graphite strip by pulsed current.

The images of the explosion of the graphite strip, at a typical time for a discharge voltage of 20 kV, is shown in Figure 3. The expansion of the graphite strips began immediately after the start of the discharge. The graphite strips contain a large amount of nitrogen gas. The electrical energy injected into the graphite strip was not sufficient to achieve a change in the state of the graphite strip. Therefore, the expansion of the strip was due to the thermal expansion of the gas. In the image taken 2.39 µs after the start of the discharge, numerous small arc-shaped shock waves are observed. This indicates that the gas inside the sample was rapidly heated, resulting in expansion and explosion. The black expansion zone blocked the light transmitted from the laser. When the electrical resistance started to increase, light emission was observed in the black expansion band (5.29 μs). When the nitrogen atom recombines, it transitions from the higher energy level B_3_Πg to the lower level A^3^Σu. This is a molecule-to-molecule transition, and the light emission spectrum is band-like (500–800 nm) [40]. It is to be noted that this wavelength region covers the band-pass filter attached to the camera lens. In other words, the light-emission region in the image shows nitrogen molecules in the transition process. In the image captured at 9.09 μs, when the energy injection was completed, the current path was observed to be occupied by nitrogen molecules. The increase in electrical resistivity can be attributed to the removal of conductive graphite from the current path. In the image captured at 11.29 µs, interference fringes of transmitted light (laser light) and shock waves are observed around the expanding gas, and the region of transmitted light is enlarged at 13.99 µs. As the transparency of water as a medium increases, the laser light is transmitted. Therefore, we can conclude that the black expansion band corresponds to exfoliated single-or few-layer graphene. A similar phenomenon was observed at an earlier time for a discharge voltage of 30 kV. We believe that the recovery of graphene increases under higher voltage conditions because the gas inside the sample can be heated and exploded more quickly. In fact, in our previous studies, the recovery of thin graphene was higher under high-voltage conditions [30]. The videos of explosion at each condition are provided in the Supplementary Materials (Videos S1 and S2).

**Figure 4:**
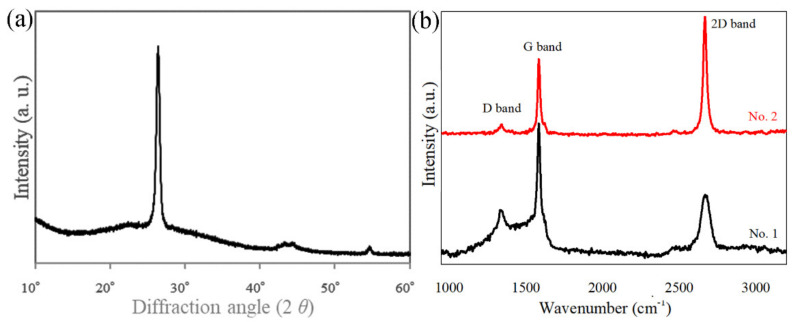
(**a**) XRD pattern of the recovered sample and (**b**) Raman spectra of graphite strip (No. 1) and recovered sample (No. 2).

The XRD pattern of the particles recovered at the discharge voltage of 30 kV is shown in Figure 4a. A sharp XRD peak appearing at 26.5° is observed, which indicates the high crystallinity of recovered graphene materials. Furthermore, Raman spectrum analysis provides a quick and facile structural and quality characterization of carbon phases [30]. Figure 4b shows three typical Raman bands of carbon materials in Raman spectra of raw graphite strip and recovered sample, such as D band (1337 cm^−1^), G band (1586 cm^−1^) and 2D band (2669 cm^−1^). The intensity ratio of 2D band to G band (I_2D_/I_G_) has been applied to identify the layers of recovered graphene samples from graphite, considering that the I_2D_/I_G_ value of graphene material is stronger than that of raw graphite [29,30]. Based on Figure 4b, the I_2D_/I_G_ values of graphite strip and recovered sample are 0.45 and 1.56, respectively. It reveals that the recovered sample is few-layer graphene [29,30].

**Figure 5:**
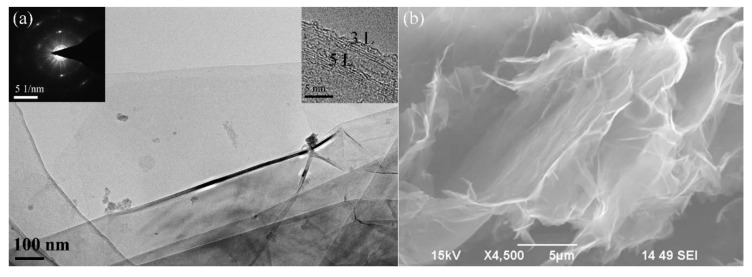
(**a**) Representative TEM image and (**b**) SEM image of recovered sample. The scale bars of SAED pattern and HRTEM image in inset of Figure 5a are 5 1/nm and 5 nm, respectively.

TEM and HRTEM images (Figure 5a) show the microstructure of sample recovered at discharge voltage of 30 kV, revealing the presence of ultra-thin, wrinkled, and extended graphene nanosheets with an interlayer distance of approximately 0.34 nm. Based on the numbers of graphitic layers in above films (see inset of Figure 5a), the recovered sample can be identified as few-layer graphene. Furthermore, the selected area electron diffraction (SAED, inset of Figure 5a) pattern of recovered sample display hexagonally arranged ring-like diffraction spots. This is in accordance with the typical result observed in few-layer graphene [29,30]. Moreover, Figure 5b presents typical SEM images of a sample recovered at discharge voltage of 30 kV, demonstrating curved and extended ultra-thin carbon films which is the typical micromorphology of graphene [29,30,31] owing to the thermodynamically instability of two-dimensional materials.

Cho et al. reported that, when a pulsed current is applied to a silicon rod with high electrical resistivity in air, the current flows through the rod surface and vaporizes part of its surface, ionizing the air around the rod and forming a plasma channel [41]. Although this study was conducted in airless water, the nitrogen gas contained inside the sample may have formed plasma channels. We concluded that the electrical resistivity properties of graphite, and the discharge in water, help to efficiently inject electrical energy into the gas contained in the graphite strips and generate shock waves for graphene exfoliation.

## 4. Conclusions

Pulsed high current experiments on graphite strips, which contain a large amount of nitrogen gas, were conducted to reveal the mechanism of graphene formation by exfoliation. The heating and explosion of the graphite strips by pulsed current were successfully observed using a high-speed video camera and by adjusting the current density conditions to prevent the graphite from undergoing a state change. The discharge voltage and the current profiles were measured synchronously with the high-speed video camera observations. Small shock waves generated by the expansion of the graphite strip and the explosion of the heated gas were observed immediately after the start of the discharge. The delaminated products were observed as turbid regions in the medium. With the same porosity of graphite strips and reduced pore size, explosions are expected to occur faster and at more locations in the sample. Hence, pore adjustment of the starting material may contribute to the production of high-quality graphene.

## Data Availability

The data presented in this study are available on request from the corresponding author.

## Supplementary Materials

The following are available online at https://www.mdpi.com/article/10.3390/nano11051223/s1, Video S1: The explosion of graphene strips at 20 kV discharge voltage condition. Video S2: The explosion of graphene strips at 30 kV discharge voltage condition.
